# The impact that family members’ health care experiences have on patients’ trust in physicians

**DOI:** 10.1186/s12913-021-07172-y

**Published:** 2021-10-19

**Authors:** Nao Oguro, Ryo Suzuki, Nobuyuki Yajima, Kosuke Sakurai, Takafumi Wakita, Mark A. Hall, Noriaki Kurita

**Affiliations:** 1 Department of Clinical Epidemiology, Graduate School of Medicine, Fukushima Medical University, Fukushima, Japan; 2 Division of Rheumatology, Department of Medicine, Showa University School of Medicine, Tokyo, Japan; 3 Center for Innovative Research for Communities and Clinical Excellence (CiRC2LE), Fukushima Medical University, Fukushima, Japan; 4 Department of Pediatrics, Niigata University Graduate School of Medical and Dental Sciences, Niigata, Japan; 5 Department of Healthcare Epidemiology, School of Public Health in the Graduate School of Medicine, Kyoto University, Kyoto, Japan; 6 Department of Pharmacy, Showa University Hospital, Tokyo, Japan; 7 Department of Sociology, Kansai University, Osaka, Japan; 8 School of Law and School of Medicine, Wake Forest University, Winston-Salem, United States North Carolina; 9 Department of Innovative Research and Education for Clinicians and Trainees (DiRECT), Fukushima Medical University Hospital, Fukushima, Japan; 10 Department of Clinical Epidemiology, Graduate School of Medicine, Fukushima Medical University, 1 Hikarigaoka, Fukushima 960-1295 Fukushima City, Japan

**Keywords:** Japan, Non-communicable disease, Medical care, Physicians, Physician trust, Dissatisfaction, Family experience

## Abstract

**Background:**

A family member’s negative experiences with medical care have long-term effects on a patient’s attitudes and emotions. However, the impact of family members’ experiences on patients’ trust in their own physicians and in physicians generally is poorly understood. This study aims to quantify these associations.

**Methods:**

A cross-sectional online survey involving adults with non-communicable diseases (cardiac disease, diabetes, cancer, depression, and rheumatic disease) was conducted in Japan during April 2020. The main exposure variable was dissatisfaction with the medical care that family members had received. The main outcomes were patients’ (*N* = 661) own trust in their personal physicians and in physicians generally. The study adopted the Japanese version of the Abbreviated Wake Forest Physician Trust Scales. Both 5-item scales (general and individual physician trust) were translated and validated for the study. The total scores were transformed into a scale of 0-100 points. A series of linear mixed-effects models with consideration for clustering effect by prefectures were fit.

**Results:**

The results showed a lower rating for trust in physicians generally as compared to trust in the respondent’s personal physician (mean 57.0 vs. 66.4 points; *p* < 0.001). Furthermore, dissatisfaction with a family member’s medical care was associated with lower trust in physicians generally (mean difference − 9.58, 95 %CI -12.4 to -6.76). Interestingly, dissatisfaction with a family member’s care was also associated with lower trust in the respondent’s personal physician (mean difference − 3.19, 95 %CI -6.02 to -0.36), but the magnitude of this association was weaker. The lower trust in personal physicians may be mediated by reduced trust in physicians generally.

**Conclusions:**

We suggest that physicians enquire about past patients’ negative experiences, including dissatisfaction with family members’ medical care, to repair hidden loss of trust, when they sense that patients doubt them or physicians generally.

**Supplementary Information:**

The online version contains supplementary material available at 10.1186/s12913-021-07172-y.

## Introduction

Among potentially modifiable patient attitudes, future expectations, or trust, toward physicians are important factors in decisions regarding treatment and continued care [1]. Trust in physicians has been demonstrated to be associated with adherence to medical treatment and continuity of follow-up [2]. There are two types of trust in physicians: trust in individual physicians (interpersonal physician trust), and in physicians generally (general physician trust) [3]. General physician trust can strongly influence the formation of interpersonal physician trust in a specific, known physician, and depends to some extent on an individual’s past experience with their personal physicians [3, 4].

Family members can evaluate the quality of medical care directly through their involvement in their children’s and parents’ medical care, [5, 6] especially in intensive or oncological care [5, 7]. Family members can also assess the quality indirectly through shared medical experiences conveyed by other family members during everyday communication. Family members’ evaluation of the quality of a patient’s medical care is likely to occur more often in Japan compared to western countries because Asian cultural values emphasize family support [8]. Medical decision making in Japan is characterized by the patient’s family involvement and indeed many patients prefer group decision making [9, 10]. Patient and family members’ satisfaction is one of the subjective quality metrics of patient expectations and preferences for medical care experienced by patients and their family members [6, 11]. This is evident from the fact that low satisfaction subsequently influences the health-behaviors of patients and/or their family members, [11] and the possibility of medical litigation claims after unfavorable outcomes [6, 12, 13]. Therefore, family members’ dissatisfaction with patients’ medical care can cause long-term harm to patient–doctor relationships, resulting not only in behavioral changes in family members but also changes in their attitude toward medical care.

For example, some bereaved children of cancer patients have a long-standing distrust toward the medical care provided to cancer patients [5]. A study involving family members of patients who had experienced medical errors found that family members reported a loss of trust in healthcare and avoidance of medical care in general [14]. This system-level loss of trust in healthcare can include a loss of general physician trust [3, 15]. Furthermore, general physician trust is said to strongly influence the formation of interpersonal physician trust.[3] However, trust in an individual physician often stays at a remarkably high level, with patients being more willing to forgive physicians they trust [3, 15]. However, these possibilities have not been studied extensively.

Trust, which is a forward-looking evaluation of an ongoing relationship, is distinguished from satisfaction, which is an assessment of a past event [16]. Thus, while it is self-evident that patients’ trust in a physician involved in a single medical service correlates with their satisfaction with the service provided, it is not fully understood whether dissatisfaction with a single medical service experience influences trust in a physician who is not involved in the service. Furthermore, little study has been done on cases where the experience was not for the patients themselves but for their family members.

**Fig. 1:**
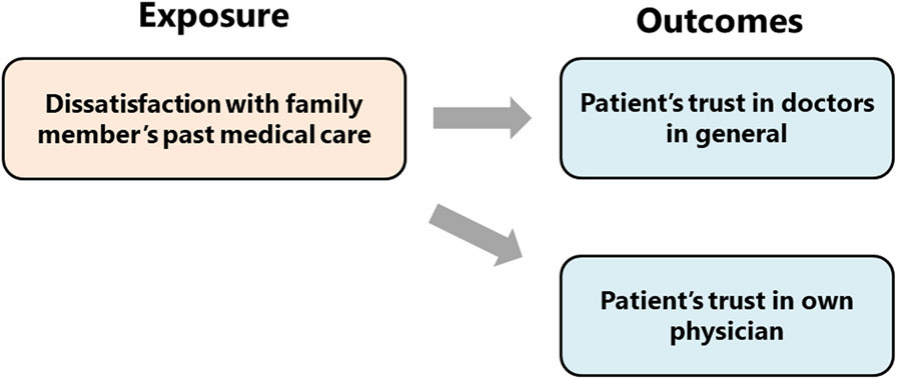
Conceptual framework for this study

In the present study, we analyzed the extent to which family members’ dissatisfactory past medical experiences influence patients’ trust in their own current physicians and physicians in general (Fig. 1). This is a critical gap in our understanding of trust. Clarification of the hidden origins of distrust will serve as a basis for building a good relationship with patients in a new medical encounter.

## Materials and methods

### Setting and selection

This cross-sectional study was approved by the Ethics Review Board of Kansai University. We used an online panel survey provided by a web-based company (Cross Marketing, Shinjuku-ku, Tokyo) to recruit Japanese participants with non-communicable diseases aged 20 years or older. The reason for the age restriction is that patients transition from pediatric care to adult primary care between 18 and 20 years of age. The sampling method is consecutive sampling from the diseases panel that the company owns. The company continued to ask the registrars on the panel until we reached our target number of respondents. Considering that there are 23 levels of variables used in the analysis of associations, and that linear regression requires a sample size of 20 people per level, we required a minimum of 460 respondents with complete data. After considering the possibility that about 10 % of the data might have missing values and that about half of the responses might be careless, we decided to collect about 1000 responses. The respondents were offered a financial incentive. They were discouraged from answering more than once, and researchers could only use their initial response. The response data was collected between April 27 and April 28, 2020.

### Demographic information

Characteristics such as age, gender, education level, total household income, and zip code were collected using self-reports. We categorized respondents’ prefectures based on the first three digits of their zip codes. The duration of the patient–physician relationship was categorized as less than 1 year, 1 to 3 years, and more than 3 years.

### Designing screening items

To prevent random variability and reliability loss through the answers of non-serious respondents, screening items were designed to exclude them from the analysis [17]. As multiple screening items are more effective than a single item, three such items were incorporated before the main survey [18].

For the first item, respondents had to select a non-communicable disease for which they had received medical treatment twice or more times within the past six months, from a set of eight options. Multiple selections were allowed. For the next item, they had to choose the illness that was most troublesome among those selected in the previous item. If they selected a different disease from the one(s) chosen previously, it meant that either the option(s) chosen from the first item or the second item would have been incorrect; thus, such participants were excluded.

Respondents were then instructed to write the name of a medication prescribed for their most troubling disease in a free-text format. The researchers searched online for label information based on the drug name provided, to assess whether the relevant disease was indicated, in which case the responses were considered valid. Otherwise, the respondents were excluded. However, respondents who chose cancer and wrote “none” for their prescribed drugs were included, as not all cancer treatments require prescribed drugs (e.g., the watch-and-wait method of care is also a reasonable care plan). Two researchers conducted these assessments independently; if the evaluations varied, decisions were reached by consensus.

**Fig. 2:**
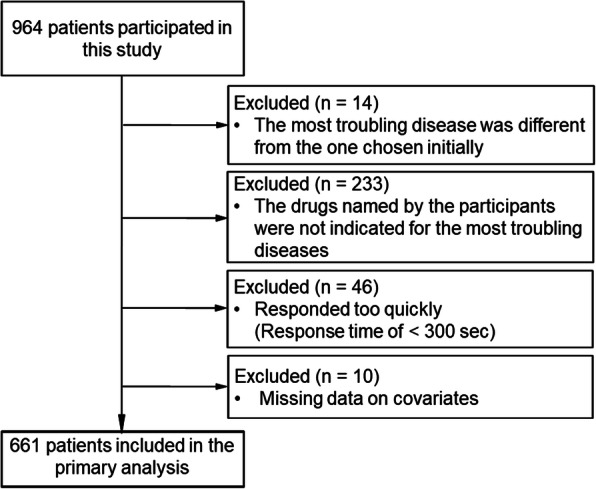
Flow of the study

Respondents were further screened using a response time cut-off since those who responded too quickly may have given spurious answers [17, 18]. Through a pilot test among researchers and their assistants, we found that at least five minutes (300 s) were required to complete the survey. Therefore, those respondents taking less than 300 s were excluded. Figure 2 provides the flow of the study design.

#### Dissatisfaction with medical care received by family

Regarding dissatisfaction with the medical care received by their family, the respondents were first given the following instructional statement: “*Please look back on the medical care your family has received. Please choose 1 (yes) if you have ever experienced the following, or 2 (no) if you have not.*” Thereafter, the following question was asked: “*Have you ever been dissatisfied with your family member’s medical care during hospitalization or hospital visits*?”

#### Wake forest physician trust scales: trust in doctors generally and interpersonal trust in physician scales

For this study, short versions of the 5-item “Trust in Doctors Generally” and “Interpersonal Trust in Physician” scales developed by Dugan and Hall, [19] were translated into Japanese. The initial translation was performed by two physicians (N.Y. and N.O.), a physician researcher (N.K.), and a quantitative psychologist (T.W.) with experience in scale development 20. Next, these translations were back translated into English by two bilingual translators (one American and one Canadian) and the wording was compared to the originals to make necessary amendments to the translation. Finally, the back-translated version was sent to the original author (Hall), and additional minor improvements were made. The final versions, approved by the original author, are shown in Supplementary Table 1 (Interpersonal Trust in a Physician, see Additional file 1) and 2 (Trust in Physicians Generally, see Additional file 2).

**Interpersonal Trust in a Physician**. Before answering the short version of the Interpersonal Trust in a Physician scale, respondents were instructed as follows: “*Please think of the doctor who cares for your [the most troublesome disease chosen by the participants was automatically displayed here] when you answer these questions. He/she will be considered your doctor for this survey. For the next questions, we are interested in your honest opinion about your doctor. Please choose the answer that best matches your thoughts for each question*.”

**Trust in Doctors Generally.** Before answering the short version of the Trust in Doctors Generally scale, respondents were instructed as follows: “*The following questions may seem similar to the previous ones. However, they are not about your doctor but doctors in general. There is no need to be concerned if you have not thought about these issues before. There is no right or wrong answer. Please choose the answer that best matches your thoughts about doctors in general.*”

For each of the five translated items in each scale, the respondents were instructed to respond on a Likert scale ranging from 1 (strongly disagree) to 5 (strongly agree). We then inverted the score for one negatively-worded item, and changed the sum of the score to a scale ranging from 0 to 100.

#### Additional attitudes

To assess additional attitudes, a self-report online questionnaire containing various items was used. All items were scored on a 5-point Likert scale ranging from 1 (strongly disagree) to 5 (strongly agree).

**Patient’s general level of interpersonal trust**. This was measured using the General Trust Scale [22]. The scale includes six items, with the sum of the items representing the scale score. We expected that both the Interpersonal Trust in a Physician and Trust in Doctors Generally scales would be associated with the General Trust Scale, but the relationships would not be strong, since general interpersonal trust has not previously been found to be strongly related to trust in physicians [2, 21 ].

**Satisfaction with doctors in general.** We assessed this with the item, “*Overall, I am very satisfied with doctors.*”^19^

**Patients’ satisfaction with their physicians.** This was assessed using the item, “*Overall, you are extremely satisfied with your doctor*.’ [19] Previous studies have strongly correlated this item with trust in a physician [19, 23, 21].

**Patient recommendations about their physicians.** This was examined using the item, “*You would recommend you r physician to your family and friends.*” [19] Higher interpersonal trust scores are expected to be correlated with a better rating of recommendation for their physicians. 

**The desire to change one’s physician.** This was examined using the item, “*I have a desire to chan ge my physician.*” [19] We expect that the lower the score on interpersonal trust, the stronger a patient’s desire to change physicians will be.

**Attitude toward adhering to physician treatment.** We used one item *(Home treatment is often better than doctor-prescribed medicine* [24]*)*from a scale that measures skepticism about medical care. [25] We considered that a low level of trust in a physician represents a stronger belief in the effectiveness of home treatment.

### Statistical analysis 

An exploratory factor analysis was done in R version 4.0.1 using the psych package. The other analyses were done in Stata/SE version 15 (Stata Corp., College Station, TX, USA). Respondents’ characteristics were summarized as means a nd standard deviations for continuous variables and frequencies and proportions for categorical variables. 

For the Interpersonal  Trust in a Physician and Trust in Doctors Generally scales, we performed exploratory factor analyses with MINRES methods to examine the factorial structures among the 10 combined items. The raw scores for reverse items were used as is. The number of latent factors was assessed by eigenvalues attenuation. [26] The absolute factor loading magnitudes were calculated. Reliability was assessed by Cronbach’s α and McDonald’s ω coefficients.[27] Furthermore, we examined the construct validity of the Interpersonal Trust in Physician scale by testing correlations between the scale and the following factors: patient’s satisfaction with their physician, patient’s recommendations of their physician, satisfaction with doctors in general, duration of the relationship with their physician, [19] and the General Trust Scale. Moreover, we explored the construct validity of the Trust in Doctors Generally scale by testing correlations between it and the Interpersonal Trust in a Physician scale, general satisfaction with doctors, attitude toward adhering to physician treatment, and the General Trust Scale. Spearman’s correlation coefficients were calculated to test these correlations. In addition, to examine whether the respondents rated Interpersonal Trust in a Physician and Trust in Doctors Generally scale items differently, a paired t-test was applied.

Furthermore, to estimate the association between respondents’ dissatisfaction with medical care received by their family and their Trust in Doctors Generally scale score, we fitted a series of linear mixed-effects models with consideration for the clustering effect by prefectures. In unadjusted analysis, only the respondent’s dissatisfaction was fit. In the multivariable-adjusted analysis, the respondent’s dissatisfaction, as well as covariates (age, gender, level of education, total household income, and comorbidities), were fitted to a single model.

Similarly, a series of linear mixed-effects models, with consideration for the clustering effect by prefectures, were fitted to examine the respondents’ Interpersonal Trust in a Physician and dissatisfaction with the medical care received by their family. In unadjusted analysis, only the respondent’s dissatisfaction was fit. In the multivariable-adjusted analyses, first, the respondent’s dissatisfaction, as well as covariates (age, gender, level of education, total household income, comorbidities, and duration of relationship between the patient and their physician), were fitted to a single model (adjusted model 1). Second, to assess whether the Trust in Doctors Generally score mediates the relationship between the respondent’s dissatisfaction and Interpersonal Trust in a Physician, covariates in adjusted model 1 plus Trust in Doctors Generally were entered in the linear mixed-effect model (adjusted model 2). These covariates were chosen as they could be associated with both patient dissatisfaction with medical care and trust in physicians.

## Results

The participant characteristics are presented in Table 1. Overall, 3,199 individuals received email invitations from the survey company to participate in the survey, and 964 did so, yielding a response rate of 30.1 %. Of those, 303 were excluded: 293 because of the three screener items, and 10 because of missing covariates. Subsequently, 661 participants (women: *N* = 175 [26.5 %]; Mean age: 62.7 ± 10.1) were included from the primary analysis. The participants’ region of residence extended to 46 prefectures, with Kanto region being the most common (41.3 %). The most common troublesome diseases were cancer (36.6 %), diabetes (26.5 %), depression (17.7 %), and heart disease (17.3 %).

**Table 1:** Participant characteristics

	Total	
	*N* = 661	
**Age, in years**	62.7	(10.1)
**Women, N(%)**	175	[26.5 %]
**Education, N(%)**		
*Junior high school*	19	[2.9 %]
*High school*	209	[31.6 %]
*Junior college*	65	[9.8 %]
*University*	325	[49.2 %]
*Graduate school*	30	[4.5 %]
*Not answered*	13	[2.0 %]
**Total household income, N(%)**		
*< 1,000,000 yen *	40	[6.1 %]
*1,000,000 – < 3,000 000 yen*	157	[23.8 %]
*3,000,000 – < 5,000,000 yen*	203	[30.7 %]
*5,000,000 – < 10,000,000 yen*	205	[31.0 %]
*10,000,000 or more yen*	56	[8.5 %]
**Region, N(%)**		
*Hokkaido*	35	[5.3 %]
*Tohoku*	34	[5.1 %]
*Chubu*	98	[14.8 %]
*Kanto*	273	[41.3 %]
*Kansai*	132	[20.0 %]
*Chugoku*	29	[4.4 %]
*Shikoku*	19	[2.9 %]
*Kyushu-Okinawa*	41	[6.2 %]
**Reported disease, N(%)**		
*Cardiac disease, arrhythmia*	37	[5.6 %]
*Cardiac disease, angina pectoris or myocardial infarction*	119	[18.0 %]
*Cardiac disease, heart failure*	15	[2.3 %]
*Diabetes*	191	[28.9 %]
*Connective tissue disease*	17	[2.6 %]
*Cancer*	255	[38.6 %]
*Depression*	127	[19.2 %]
**The most troublesome disease, N(%)**		
*Cardiac disease, arrhythmia*	17	[2.6 %]
*Cardiac disease, angina pectoris or myocardial infarction*	89	[13.5 %]
*Cardiac disease, heart failure*	8	[1.2 %]
*Diabetes*	175	[26.5 %]
*Connective tissue disease*	13	[2.0 %]
*Cancer*	242	[36.6 %]
*Depression*	117	[17.7 %]
**Duration with patients’ physician, N(%)**		
*< 1 year*	60	[9.1 %]
*1 – <3 years*	212	[32.1 %]
*≥ 3 years*	389	[58.9 %]

Continuous variables summarized as mean and standard deviation (in parentheses)

Categorical variables summarized as frequency and proportion (in square brackets)

### Interpersonal trust in a physician scale and the trust in doctors generally scale

The eigenvalue attenuation (5.31, 1.37, and 1.05 for the first, second, and third factors, respectively; Supplementary Fig. 1, see Additional file 3) suggested a two-factor solution for the combined 10 items. The absolute values of the factor loadings for items 1 to 5 ranged from 0.44 to 0.86 in factor 1, all of which were above 0.4 (Supplementary Tables 3, see Additional file 4). The absolute values of the factor loadings for items 6 to 10 ranged from 0.42 to 0.89 in factor 2, all of which were above 0.4 (Supplementary Tables 3, see Additional file 4). The inter-factor correlation was moderate (*r* = 0.64). No double loadings between factors occurred for any of the items. Thus, items 1 to 5 could be included in a single factor and reasonably constitute the Japanese version of the Trust in Doctors Generally scale, whereas items 6 to 10 could be included in another single factor and reasonably constitute the Japanese version of the Interpersonal Trust in a Physician scale. Cronbach’s alpha coefficient and McDonald’s omega coefficient were 0.85 and 0.88, respectively, for the Japanese version of the Interpersonal Trust in a Physician scale and 0.88 and 0.93, respectively, for the Japanese version of the Trust in Doctors Generally scale.

For the Interpersonal Trust in a Physician scale (mean: 66.4 ± 17.8), the scores were distributed from 0 to 100, with only 0.2 % and 5.3 % of them being at the floor and ceiling scores, respectively. As expected, construct validity was supported by the finding that the Interpersonal Trust in a Physician scale was strongly correlated with satisfaction with the physician (*ρ* = 0.724) and recommending the physician (*ρ* = 0.678), while it was strongly negatively correlated with the desire to change physicians (*ρ* = -0.632) (Table 2). Furthermore, this scale was moderately correlated with satisfaction with doctors in general (*ρ* = 0.550), with a weaker magnitude than that of the correlation between the scale and satisfaction with their physician. The scale was weakly correlated with general interpersonal trust, suggesting that the scale measured a different concept. The scale was not correlated with the duration of the relationship with the physician.

**Table 2:** Correlation between interpersonal trust in patient’s physician and selected variables

	Correlation coefficients^a^	*p*-value
**Patient’s satisfaction with the physician**	0.724	< 0.001
**Patients’ recommendations of their physicians**	0.678	< 0.001
**General satisfaction with physicians in general**	0.550	< 0.001
**Patients’ desire to change their physicians**	-0.632	< 0.001
**Duration of relationship**	0.047	0.226
**Patient’s general level of interpersonal trust**	0.243	< 0.001

^a^All variables were tested by the Spearman correlation coefficient

For the Trust in Doctors Generally scale (mean: 57.0 ± 18.4), the scores were distributed from 0 to 100, with only 0.2 % and 2.1 % of them being at the floor and ceiling scores, respectively. As expected, construct validity was supported by the finding that the Trust in Doctors Generally scale was moderately correlated with satisfaction with doctors in general (*ρ* = 0.568) (Table 3). The scale was only moderately correlated with the Interpersonal Trust in Physicians (*ρ* = 0.571) scale, and weakly correlated with general interpersonal trust (*ρ* = 0.313), suggesting that the scale measured different concepts than these. Furthermore, this scale was weakly negatively correlated with the attitude toward adhering to physician treatment (*ρ* = -0.213). The scale score was lower than the Interpersonal Trust in a Physician score (*p* < 0.001).

**Table 3:** Correlation between trust in doctors generally and selected variables

	Correlation coefficients^a^	*p*-value
**Interpersonal trust in patient’s physician**	0.571	< 0.001
**General satisfaction with physicians in general**	0.568	< 0.001
**Believes in home remedies rather than medications prescribed by doctors**	-0.213	< 0.001
**Patient’s general level of interpersonal trust**	0.313	< 0.001

^a^All variables were tested by the Spearman correlation coefficient

### Dissatisfaction with family members’ medical care: relationship with interpersonal trust in a physician and general physician trust

**Fig. 3:**
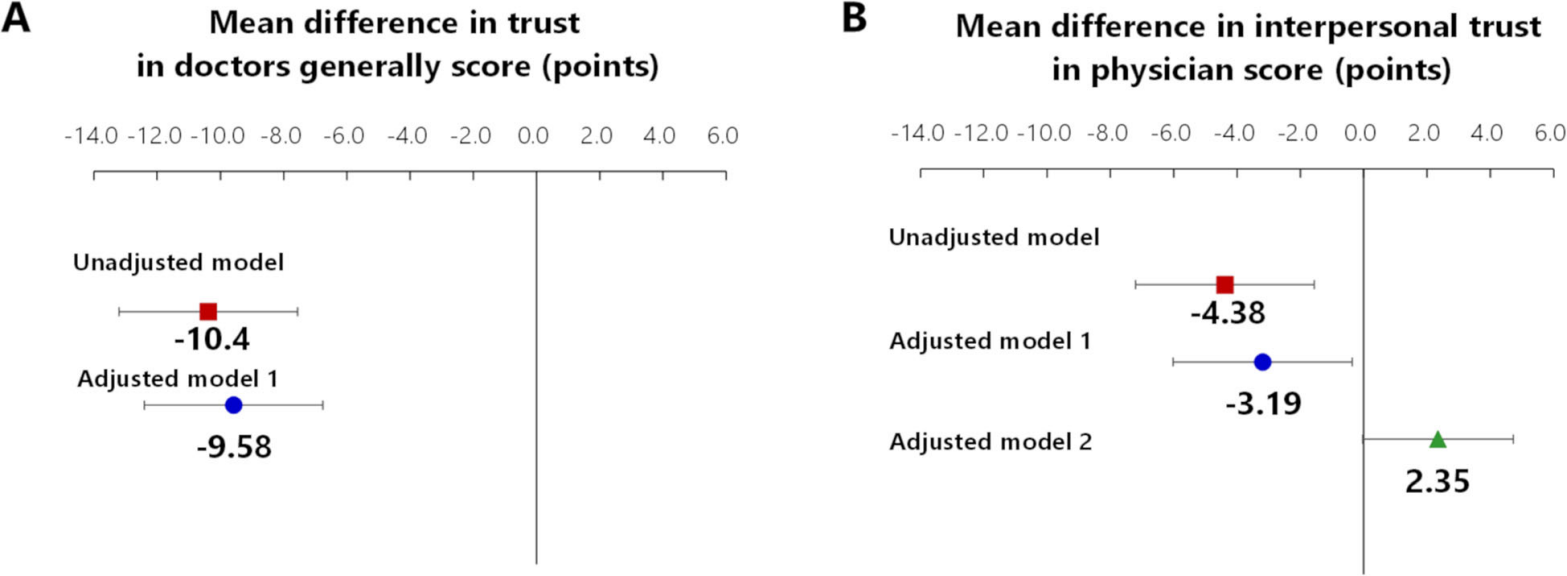
Associations of dissatisfaction with family members’ medical care with the Trust in Doctors Generally score and the Interpersonal Trust in Physician score. Mean differences were estimated using a linear mixed effect model with consideration for prefectural-level correlation using cluster variance. (**A**) Adjusted analysis 1 included age, sex, duration of patient–physician relationship, comorbidities, education, and total household income. (**B**) Adjusted analysis 1 included age, sex, duration of patient–physician relationship, comorbidities, education, and total household income. Adjusted analysis 2 included covariates included in adjusted analysis 1 and Trust in Doctors Generally score. Solid squares, circles, and a triangle indicate point estimates. Error bars indicate 95 % CIs.

Overall, 233 respondents (35.2 %) felt dissatisfaction with family members’ medical care. The association between dissatisfaction with family members’ medical care and general physician trust is shown in Table 4. Dissatisfaction with family members’ medical care was negatively associated with lower trust (mean difference − 9.58 [corresponding standardized effect size: -0.52],[28] 95 % confidence interval {CI} [-12.4 to -6.76]; Fig. 3 A, adjusted model 1). Older respondents had higher trust scores than did younger respondents (mean difference per 10-year difference: 1.92, 95 %CI [0.44 to 3.39]). Those with graduate school education had lower trust than compared to those with junior high school education (mean difference: -10.3, 95 %CI [-20.4 to -0.17]).

**Table 4:** Associations between dissatisfaction with medical care provided to patients’ families with Trust in Doctors Generally

Trust in Doctors Generally score, points	Mean difference	(95 %CI)		*p*-value
**Unadjusted model**^**a**^				
**Dissatisfaction with medical care provided to a patient’s family**	**-10.4**	**(-13.2 to -7.56)**		**< 0.001**
**Multivariable-adjusted model**^**b**^				
**Dissatisfaction with medical care provided to a patient’s family**	**-9.58**	**(-12.4 to -6.76)**		**< 0.001**
**Age, per 10 yr**	**1.92**	**(0.44 to 3.39)**		**0.011**
Sex, female	-0.81	(-4.34 to 2.73)		0.654
Education				
*Junior high school*	Ref			
*High school*	-5.29	(-13.4 to 2.85)		0.203
*Junior college*	-2.92	(-12.0 to 6.12)		0.527
*University*	-6.69	(-14.8 to 1.41)		0.105
***Graduate school***	**-10.3**	**(-20.4 to -0.17)**		**0.046**
*Not answered*	-4.94	(-17.2 to 7.34)		0.430
Total household income				
*< 1,000,000 yen*	-6.16	(-13.4 to 1.04)		0.094
*1,000,000 – < 3,000,000 yen*	-0.05	(-5.5 to 5.42)		0.987
*3,000,000 – < 5,000,000 yen*	-0.47	(-5.7 to 4.76)		0.860
*5,000,000 – < 10,000,000 yen*	-1.56	(-6.7 to 3.58)		0.552
*10,000,000 or over yen*	Ref			

Analysis of 661 patients in 46 prefectures

^a^Linear mixed effect models with consideration for prefectural level correlation

^b^Linear mixed effect model adjusted for age, sex, comorbidities, education, and total household income with consideration for prefectural-level correlation

The association between dissatisfaction with family members’ medical care and interpersonal trust in a physician is shown in Table 5. Similarly, to general physician trust, while dissatisfaction with family members’ medical care was negatively associated, the magnitude of the association was very weak (mean difference: -3.19 [corresponding standardized effect size: -0.18],[28] 95 %CI [-6.02 to -0.36]; Fig. 3B, adjusted model 1). However, the inverse association between dissatisfaction with family members’ medical care and interpersonal trust in a physician disappeared when it was further adjusted by trust in physicians generally (mean difference: 2.35, 95 %CI [-0.03 to 4.73]; Fig. 3B, adjusted model 2). In this model, respondents who reported general physician trust also had higher trust in their current physicians (mean score difference in Interpersonal Trust in a Physician score per 10-point difference in Trust in Doctors Generally is 5.79 [standardized effect size 0.33], [28] 95 %CI [5.17 to 6.42]).

**Table 5:** Associations between dissatisfaction with medical care provided to patients’ families with Interpersonal Physician Trust

Interpersonal Trust in a Physician score, points	Mean difference	(95 %CI)		*p*-value
**Unadjusted**^**a**^				
**Dissatisfaction with medical care provided to a patient’s family**	**-4.38**	**(-7.21 to -1.56)**		**0.002**
**Multivariable-adjusted**^**b**^				
**Dissatisfaction with medical care provided to a patient’s family**	**-3.19**	**(-6.02 to -0.36)**		**0.027**
Age, per 10 yr	1.13	(-0.35 to 2.60)		0.135
Sex, female	0.67	(-2.87 to 4.22)		0.709
Duration with patients’ physician				
*< 1 yr*	-2.68	(-7.5 to 2.12)		0.274
*1 – < 3 yr*	-2.61	(-5.6 to 0.40)		0.089
*3 or over yr*	Ref			
Education				
*Junior high school*	Ref			
*High school*	-4.19	(-12.3 to 3.96)		0.314
*Junior college*	2.31	(-6.7 to 11.4)		0.616
*University*	-2.85	(-10.9 to 5.25)		0.491
*Graduate school*	-7.99	(-18.1 to 2.12)		0.121
*Not answered*	-2.02	(-14.3 to 10.3)		0.748
Total household income				
*< 1,000,000 yen*	-6.99	(-14.2 to 0.21)		0.057
*1,000,000 – < 3,000,000 yen*	-1.43	(-6.9 to 4.04)		0.608
*3,000,000 – < 5,000,000 yen*	-2.61	(-7.9 to 2.64)		0.330
*5,000,000 – < 10,000,000 yen*	-2.67	(-7.8 to 2.47)		0.309
*10,000,000 or over yen*	Ref			

Analysis of 661 patients in 46 prefectures

^a^Linear mixed effect models with consideration for prefectural level correlation

^b^Linear mixed effect model adjusted for age, sex, duration with patients’ physician, comorbidities, education, and total household income with consideration for prefectural-level correlation

## Discussion

We examined whether, among patients with non-communicable diseases, dissatisfaction with their family members’ medical care was associated with lower trust in physicians generally, as well as in the patients’ own physicians. Past experience of dissatisfaction with family members’ care was associated with a greater reduction in the patients’ general physician trust than in trust in the patients’ own physicians. In addition, our study suggests that the lower trust in their physicians may be mediated by lower trust in physicians generally. Our findings highlight the importance of researching dissatisfaction with family members’ medical care to identify hidden sources of lost trust in physicians.

In particular, our findings corroborate previous studies and promote insight into trust in physicians. First, a previous study involving the bereaved children of cancer patients revealed long-lasting distrust of the cancer-stricken parents’ medical care among some individuals. [5] However, that study did not examine whether dissatisfaction with the family’s medical care resulting from poor outcomes lowered the children’s own trust in the children’s current physicians. Another study that included the family members of patients who had suffered medical errors described a decrease in the family member’s trust in healthcare at the time, but the study did not quantify the extent to which such negative past experiences affected the family member’s trust in their current physicians and physicians in general.[14] Second, whereas previous reports have indicated that physicians’ image is generally constructed by the media and informal public opinion, [1, 3] we were able to show, for the first time, that the individual experience of dissatisfaction with a family member’s medical care is an important factor in reducing the individual’s trust in general physicians. Third, our finding that dissatisfaction is associated with a milder decline in trust toward personal physicians than toward physicians in general confirms that interpersonal physician trust is more resilient than trust in the medical profession generally. [3, 15] Last, we found that, among Japanese respondents diagnosed with chronic diseases, interpersonal physician trust was rated higher than trust in physicians in general. This supports the findings of an American study involving a mostly healthy general population. [3] This indicates that these trust measures are useful across disease types and countries.

Our findings could be useful for physicians and researchers in several ways. First, doctors should consider whether their patients have had any negative medical experiences. This includes dissatisfaction with family member’s medical care, especially if the patient expresses skepticism toward general medical care or the proposed treatment plan. In doing so, concerns can be addressed. Although the sources of dissatisfaction with family members’ medical care may be broad, including those not attributable to physicians, current physicians can ask about attitudes attributable to past physicians in particular—examples include inquiring about the suitability of the family member’s treatment, [29] treatment outcome, [29] physician’s kindness, [11, 12] sufficient time with physicians, [12, 30] or participation in decision making. [30] During these discussions, the doctor should convey compassion, assure patients that not all medical staff are alike, and aim to not disappoint them again. After allowing the patient to share the past problem by expressing their anger or anxiety, the doctor should attempt to rebuild a new patient–physician relationship. In particular, active listening and empathy could restore general trust in physicians and strengthen patients’ trust in their current physicians. This was evident in a training program for physicians that focused on communication skills which showed an increase in patients’ satisfaction [31] as increased satisfaction is likely to foster patients’ trust in clinical encounters. [16] Alternatively, it is possible that facilitating conversations about these dissatisfactory medical experiences by other health professionals, such as medical social workers, could reduce current trust in physicians and this would be worthy of study. Second, we found that the magnitude of patients’ lower trust associated with past dissatisfaction with their family members’ medical care is greater in the case of physicians in general than their own physicians. This may reinforce the pathways of dissatisfaction with family members’ medical care discussed in a previous study. [32] Initially, family members may naïvely trust medical professionals to take care of their relative’s illness regardless of the accompanying day-to-day challenges. [32] However, the reality of medical care, which, for example, might mean focusing on the disease rather than on their relative as a person, may cause conflict and potential long-term loss of the family members’ trust in physicians in general. Alternatively, less impairment of trust in physicians that patients know personally from previous negative medical experiences, than trust in physicians generally, may be attributed to the actions taken by personal physicians to more directly foster interpersonal trust during their practice. [1] Third, we found that lower general physician trust may mediate lower trust in current physicians associated with dissatisfaction with family members’ medical care. This supports the notion that trust in physicians generally can influence the formation of interpersonal physician trust. [3, 4]

Our study has several strengths. First, we examined the validity and association among patients with a variety of chronic diseases, including heart disease, diabetes, depression, connective tissue disease, and malignancy. Therefore, our findings about trust in physicians can be applied to a variety of disease settings. Second, by simultaneously conducting a psychometric analysis (i.e., factor analysis) of trust in patients’ physicians and trust in physicians generally, we showed that the concepts of each scale are distinct. Third, we demonstrated for the first time that the mechanism of trust in both individual physicians and physicians in general is similar between the United States and Japan, despite notable differences between these two settings. In Japan, for instance, unlike the United States, all citizens are covered by universal health insurance and have unlimited access to physicians. Thus, our findings support the understanding that both concepts of trust have universal features.

Several limitations of this study warrant a mention. Our study population may not be representative of patients with the same non-communicable diseases because our survey was based on sampling from the panel data that the company registered. However, we believe that this does not affect the associations between dissatisfaction with family members’ medical care and trust in physicians. Furthermore, the non-communicable diseases surveyed were based on self-reports and may not have been correctly identified. However, by ascertaining the drug names provided by the respondents and cross-checking them against the chosen diseases, we verified the truthfulness of the diseases that were reported. Another limitation relates to the fact that we did not investigate the reasons for dissatisfaction with family members’ medical care. Thus, the mechanism of lower trust in physicians associated with dissatisfaction could be explored in qualitative studies. Beyond physicians, other factors—including nurses’ care, hospital waiting time, and hygiene—could also influence satisfaction.[29] These may be considered in future research.

## Conclusions

In summary, dissatisfaction with family members’ medical care was associated with lower trust among patients in their current physician and physicians generally. The magnitude of lowered trust was greater for physicians in general than for current physicians. Furthermore, the lower trust in current physicians could be mediated by lower trust toward physicians in general. Future research could explore interventions to restore the loss of trust in physicians arising from the dissatisfaction with past medical experiences, including negative experiences within the family.

## Supplementary information

**Additional file 1.Supplementary Table 1.** Japanese version of the Interpersonal Trust in Physician Scale

**Additional file 2: Supplementary Table 2.** Japanese version of the Trust in Doctors Generally Scale

**Additional file 3: Supplementary Fig. 1.** Scree plot for the eigenvalues using the response to the combined 10 items of the Interpersonal Trust in a Physician scale and the Trust in Doctors Generally scale

**Additional file 4: Supplementary Table 3.** Descriptive statistics and factor loadings of the combined 10 items of the Interpersonal Trust in a Physician scale and the Trust in Doctors Generally scale

## Data Availability

The datasets used and/or analyzed during the current study are available from the corresponding author on reasonable request.

## Abbreviations

CI: confidence interval
