# Supplementary material for: The impact that family members’ health care experiences have on patients’ trust in physicians
Source: BMC Health Serv Res. 2021 Oct 19;21:1122. doi: 10.1186/s12913-021-07172-y (PMC8527743; doi:10.1186/s12913-021-07172-y)
Supplement: Supplementary file 1 — Additional file 1.Supplementary Table 1. Japanese version of the Interpersonal Trust in Physician Scale [file 12913_2021_7172_MOESM1_ESM.docx]

**Supplementary Table 1 Japanese version of the Interpersonal Trust in Physician Scale**

| Instruction sentences | 過去2年間に何人かの医師に診察してもらった場合、その中でもあなたが最もよく知っている医師のことを考えてこの調査に回答してください。  　過去2年間で少なくとも2回以上診察してもらった医師のことを、あなたの医師と呼ぶことにします。  　次の質問では、あなたの医師についてあなたが持っている本音の意見に関心があります。それぞれの質問について、あなたが思う程度を教えてください。*  (Original: “If you have several doctors or health professionals that you have seen in the past two years, think of the doctor (or health professional) you know the best when answering the survey. Alternatively, think of the doctor (or health professional) you have seen at least twice in the past two years—we’ll just call this person “your doctor.” For the next questions, we are interested in your honest opinion about your doctor. For each of these questions, please tell me whether you strongly agree, agree, are neutral, disagree, or strongly disagree.”) |
| --- | --- |
| Question 1 | あなたの医師はときどき, あなたの治療に必要なことよりも自身の都合を気にかける。  (Original: “Sometimes, your doctor cares more about what is convenient for (him/her) than about your medical needs.”) |
| Question 2 | あなたの医師は診療のあらゆる面で漏れがなく、きわめて注意深い。  (Original: “Your doctor is extremely thorough and careful.”) |
| Question 3 | あなたは、あなたの医師が判断した治療が最善なものであると完全に信用している。  (Original: “You completely trust your doctor’s decisions about which medical treatments are best for you.”) |
| Question 4 | あなたの医師はあなたの病気に使えるすべての治療の選択肢を率直に話す。  (Original: “Your doctor is totally honest in telling you about all of the different treatment options available for your condition.”) |
| Question 5 | 大体において，あなたはあなたの医師を完全に信頼している。  (Original: “All in all, you have complete trust in your doctor.”) |
| Response options for Questions | 全くそう思わない/そう思わない/どちらともいえない/そう思う/とてもそう思う  (Original: strongly disagree/disagree/neutral/agree/strongly agree) |

The original English version (1) is also provided for each item and response. Before using this instrument, please register through <https://noriaki-kurita.jp/resources/trust-in-physician-jpn/>. In addition, please cite this article as a reference:

Oguro N, Suzuki R, Yajima N, Sakurai K, Wakita T, Hall MA, Kurita N. The impact that family members' health care experiences have on patients' trust in physicians. BMC Health Serv Res. 2021 doi:10.1186/s12913-021-07172-y

*The instructional statements generated through the formal process of translation are presented. Modified instructional statements were used in the online survey of this study as follows: “*Please answer this section of the survey while thinking about the doctor who is taking care of your [the most troublesome disease chosen by the participants was automatically displayed in this place]. We will refer to him/her as your doctor. The following questions concern your feelings about your doctor. Please choose the answer that best matches your thoughts for each question.*”

**Reference**

1. Dugan E, Trachtenberg F, Hall MA. Development of abbreviated measures to assess patient trust in a physician, a health insurer, and the medical profession. BMC Health Serv Res. 2005;5(1):64.
