# Supplementary material for: The impact that family members’ health care experiences have on patients’ trust in physicians
Source: BMC Health Serv Res. 2021 Oct 19;21:1122. doi: 10.1186/s12913-021-07172-y (PMC8527743; doi:10.1186/s12913-021-07172-y)
Supplement: Supplementary file 2 — Additional file 2: Supplementary Table 2. Japanese version of the Trust in Doctors Generally Scale [file 12913_2021_7172_MOESM2_ESM.docx]

**Supplementary Table 2 Japanese version of the Trust in Doctors Generally Scale**

| Instruction sentences | 次の質問は、前の方でおたずねした質問に似ているように思えるかもしれません。しかし、次の質問はあなたの医師についてではなく、医師全般についておたずねするものです。  　このような問題について以前考えたことがなかったかもしれませんが、それは気にしないでください。正しい答えや誤った答えはありません。  　医師全般に関する次の文章について、あなたが思う程度を教えてください。*  (Original: “These next questions may sound similar to the ones I asked you before. However, these are about DOCTORS IN GENERAL, not your doctor. You may not have thought about these issues before, but do not worry; there are no right or wrong answers. How much do you agree or disagree with the following statements about DOCTORS IN GENERAL?”) |
| --- | --- |
| Question 1 | 医者はときどき, 患者の治療に必要なことよりも自身の都合を気にかけている．  (Original: “Sometimes doctors care more about what is convenient for them than about their patients’ medical needs.”) |
| Question 2 | 医者は診療のあらゆる面で漏れがなく、きわめて注意深い。  (Original: “Doctors are extremely thorough and careful.”) |
| Question 3 | あなたは、医者が判断した治療が最善なものであると完全に信用している。  (Original: “You completely trust doctors’ decisions about which medical treatments are best.”) |
| Question 4 | 医者は何かをごまかすようなことは決してしないだろう。  (Original: “A doctor would never mislead you about anything.”) |
| Question 5 | 大体において，あなたは医者を完全に信頼している。  (Original: “All in all, you trust doctors completely.”) |
| Response options for Question | 全くそう思わない/そう思わない/どちらともいえない/そう思う/とてもそう思う  (Original: strongly disagree/disagree/neutral/agree/strongly agree) |

The original English version (1) is also provided for each item and response.

Before using this instrument, please register through <https://noriaki-kurita.jp/resources/trust-in-physician-jpn/>. In addition, please cite this article as a reference:

Oguro N, Suzuki R, Yajima N, Sakurai K, Wakita T, Hall MA, Kurita N. The impact that family members' health care experiences have on patients' trust in physicians. BMC Health Serv Res. 2021. doi:10.1186/s12913-021-07172-y

*The instructional statements generated through the formal process of translation are presented. Modified instructional statements were used in the online survey of this study as follows: “*The following questions may seem similar to the previous ones. However, they are not about your doctor but doctors in general. There is no need to be concerned if you have not thought about these issues before. There is no right or wrong answer. Please choose the answer that best matches your thoughts about doctors in general*.”

**References**

1. Dugan E, Trachtenberg F, Hall MA. Development of abbreviated measures to assess patient trust in a physician, a health insurer, and the medical profession. BMC Health Serv Res. 2005;5(1):64.
