# Supplementary material for: The impact that family members’ health care experiences have on patients’ trust in physicians
Source: BMC Health Serv Res. 2021 Oct 19;21:1122. doi: 10.1186/s12913-021-07172-y (PMC8527743; doi:10.1186/s12913-021-07172-y)

**Supplementary Figure 1 Scree plot for the eigenvalues using the combined responses to items of the Interpersonal Trust in a Physician scale and the Trust in Doctors Generally scale**

The eigenvalue attenuation was largest between the first and second factors (5.31, 1.37, and 1.05 for the first, second, and third factors, respectively), indicating that the items had two dimensions.


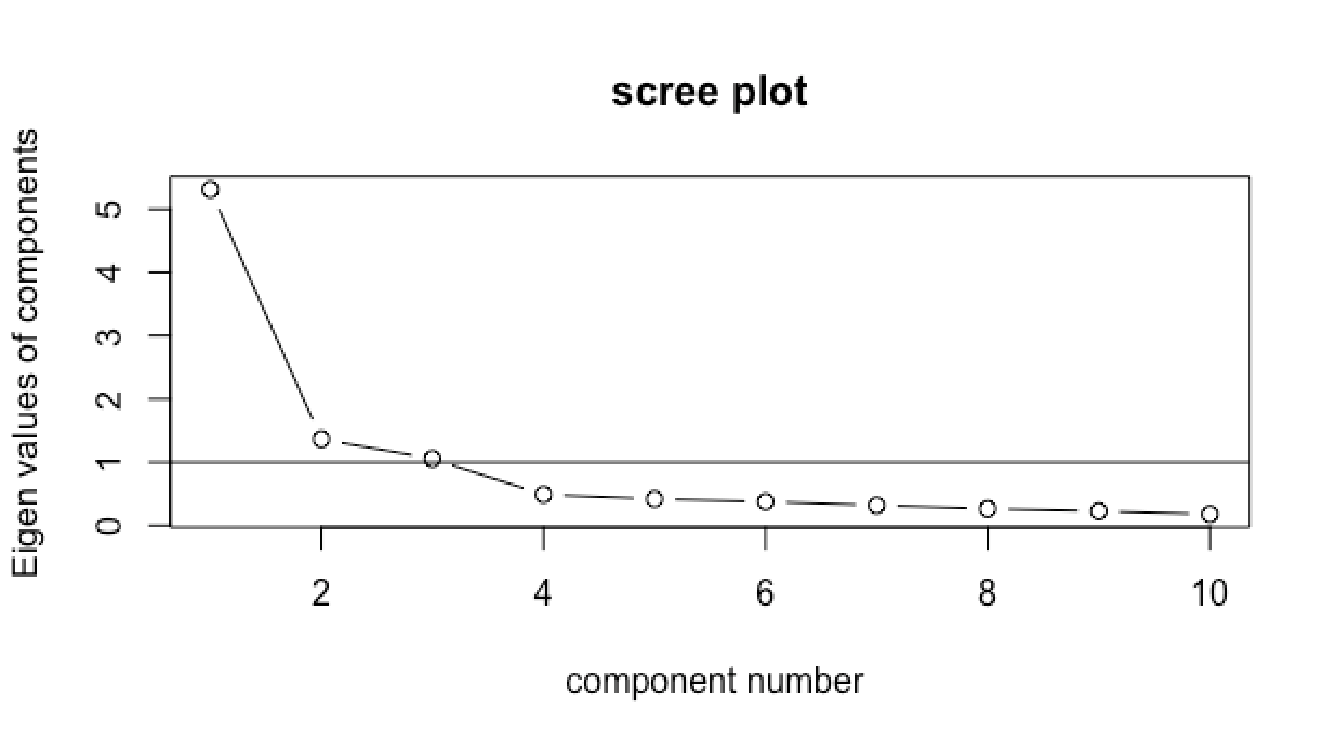

Supplement: Supplementary file 3 — Additional file 3: Supplementary Fig. 1. Scree plot for the eigenvalues using the response to the combined 10 items of the Interpersonal Trust in a Physician scale and the Trust in Doctors Generally scale [file 12913_2021_7172_MOESM3_ESM.docx]
