# Supplementary material for: The impact that family members’ health care experiences have on patients’ trust in physicians
Source: BMC Health Serv Res. 2021 Oct 19;21:1122. doi: 10.1186/s12913-021-07172-y (PMC8527743; doi:10.1186/s12913-021-07172-y)
Supplement: Supplementary file 4 — Additional file 4: Supplementary Table 3. Descriptive statistics and factor loadings of the combined 10 items of the Interpersonal Trust in a Physician scale and the Trust in Doctors Generally scale [file 12913_2021_7172_MOESM4_ESM.docx]

**Supplementary Table 3 Descriptive statistics and factor loadings of the combined items of the Interpersonal Trust in a Physician scale and the Trust in Doctors Generally scale**

|  |  |  |  |  | Factor loading | |
| --- | --- | --- | --- | --- | --- | --- |
| Item No. | Origin | Questions | Mean | SD | Factor 1 | Factor 2 |
| 1 | g4 | 医者は何かをごまかすようなことは決してしないだろう | 3.25 | 0.91 | 0.86 | -0.11 |
| 2 | g3 | あなたは、医者が判断した治療が最善なものであると完全に信用している | 3.32 | 0.89 | 0.86 | 0.05 |
| 3 | g5 | 大体において、あなたは医者を完全に信頼している | 3.43 | 0.88 | 0.85 | 0.03 |
| 4 | g2 | 医者は診療のあらゆる面で漏れがなく、きわめて注意深い | 3.18 | 0.89 | 0.82 | 0.01 |
| 5 | g1 | 医者はときどき、患者の治療に必要なことよりも自身の都合を気にかけている | 2.78 | 0.92 | -0.44 | -0.08 |
| 6 | i5 | 大体において、あなたはあなたの医師を完全に信頼している | 3.74 | 0.92 | -0.03 | 0.89 |
| 7 | i2 | あなたの医師は診療のあらゆる面で漏れがなく、きわめて注意深い | 3.47 | 0.89 | 0.08 | 0.78 |
| 8 | i3 | あなたは、あなたの医師が判断した治療が最善なものであると完全に信用している | 3.68 | 0.88 | 0.11 | 0.76 |
| 9 | i4 | あなたの医師はあなたの病気に使えるすべての治療の選択肢を率直に話す | 3.75 | 0.85 | 0 | 0.74 |
| 10 | i1 | あなたの医師はときどき、あなたの治療に必要なことよりも自身の都合を気にかける。 | 2.36 | 0.98 | 0.03 | -0.42 |

The absolute values of factor loadings for items no. 1 to no. 5 ranged from 0.44 to 0.86 in factor 1, all of which were above 0.4. The

absolute values of factor loadings for items no. 6 to no. 10 ranged from 0.42 to 0.89 in factor 2, all of which were above 0.4. The

inter-factor correlation was moderate (r = 0.64). In each item, no double loadings between factor 1 and factor 2 occurred. Thus, items

no. 1 to no. 5 could be included in a single factor and reasonably constitute the Japanese version of the Trust in Doctors Generally

scale, whereas items no. 6 to no. 10 could be included in another single factor and reasonably constitute the Japanese version of the

Interpersonal Trust in a Physician scale.
